# Failure of a “foolproof” pin-index medical pipeline system

**DOI:** 10.1186/s40981-016-0044-7

**Published:** 2016-08-08

**Authors:** Tatsuhiro Ishimura, Yoshihiro Ikuta, Tatsuo Yamamoto

**Affiliations:** Surgical Center, Kumamoto University Hospital, 1-1-1 Honjo, Chuo-ku, Kumamoto, 860-8556 Japan

**Keywords:** Medical gas, Foolproof, Misconnection

## Abstract

**Background:**

The pin-index medical gas pipeline system, which complies with Japan Industrial Standard (JIS), is considered to be “foolproof” and is widely used in Japan to avoid medical gas misconnections.

**Case presentation:**

The wall-mounted gas outlet used in our hospital (NSV outlet, CENTRAL UNI, Co., Ltd., Tokyo, Japan) contains multiple sockets, which connect to hoses with gas-specific pins. Each socket is covered with a gas-specific plastic pin guide, which is considered to make the system foolproof; i.e., to prevent misconnections. However, while checking an anesthesia machine in accordance with the guidelines developed by the Japanese Society of Anesthesiologists, an anesthesiologist found that one of the gas-specific plastic pin guides covering the wall-mounted gas outlets had disappeared; and hence, the gas outlet system was no longer foolproof. A subsequent verification test performed by engineers of the system’s manufacturer revealed that the plastic pin guides could be dislodged by applying 29.4 N of vertical force.

**Conclusions:**

It is important to check that gas outlet systems are functioning in a gas-specific manner when they are used for clinical purposes.

## Background

Medical gas misconnections can have direct life-threatening effects. Thus, safe and secure medical gas supply systems are required to ensure the safety of clinical work. In the USA, the Diameter Index Safety System (DISS) has been widely used to avoid medical gas misconnections. In Japan, the pin-index medical gas pipeline system, which complies with the Japan Industrial Standard (JIS) (product standard number: JIS T 7101) [1], has been developed. This system is considered to be “foolproof” and is designed to prevent gas-specific hoses from being connected to the wrong outlet. Each of the sockets in the wall-mounted gas outlet system contains two or three gas-specific connector holes and is connected to a hose with two or three gas-specific pins. The gas specificity of the sockets is determined by the gas-specific central angles of the hose pins and connector holes. For example, the oxygen-specific pins and holes produce a central angle of 180°, whereas the nitrous oxide-specific pins and holes exhibit a central angle of 135°.

## Case presentation

**Fig. 1:**
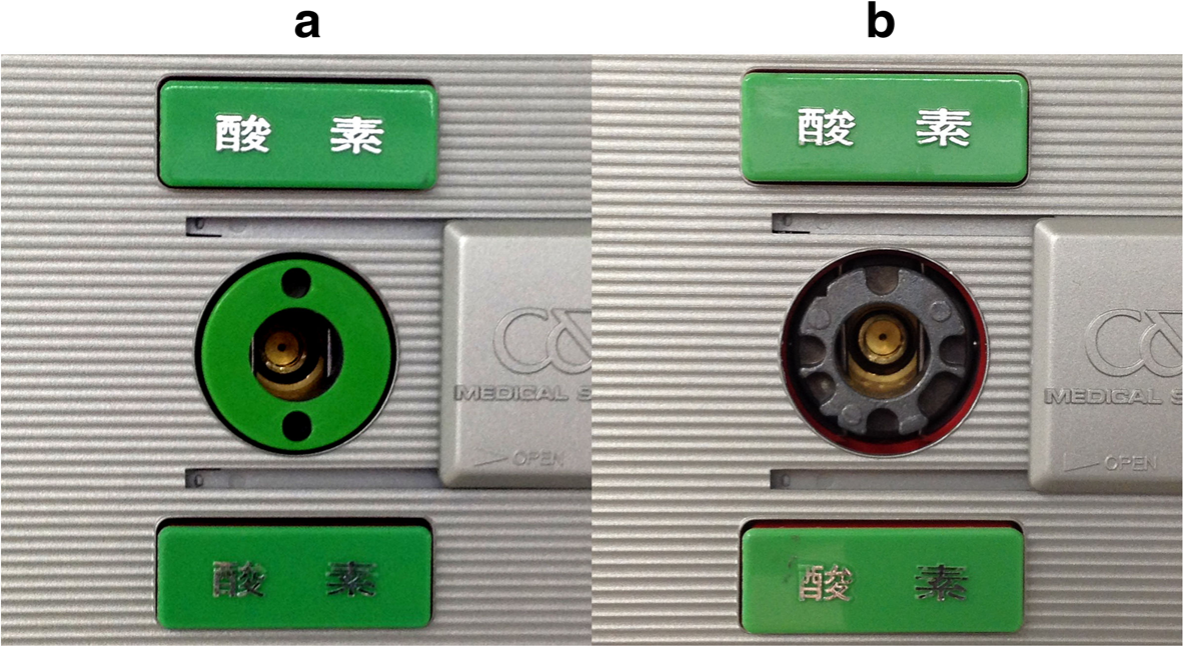
A normal oxygen socket (**a**) and the broken oxygen socket (**b**). The bare metal base block behind the pin guide contained multiple indentations. Therefore, it was possible to plug different kinds of hoses into the bare socket

In our hospital, the NSV outlet system (CENTRAL UNI, Co., Ltd., Tokyo, Japan), a pin-index medical gas pipeline system, is used. In this system, the wall-mounted gas outlet system is made of metal (zinc alloy die casting) and contains multiple indentations, each of which acts as a universal gas outlet, rather than a gas-specific outlet. Therefore, each socket is covered with a gas-specific plastic pin guide, which makes the sockets gas-specific (Fig. 1a, b).

**Fig. 2:**
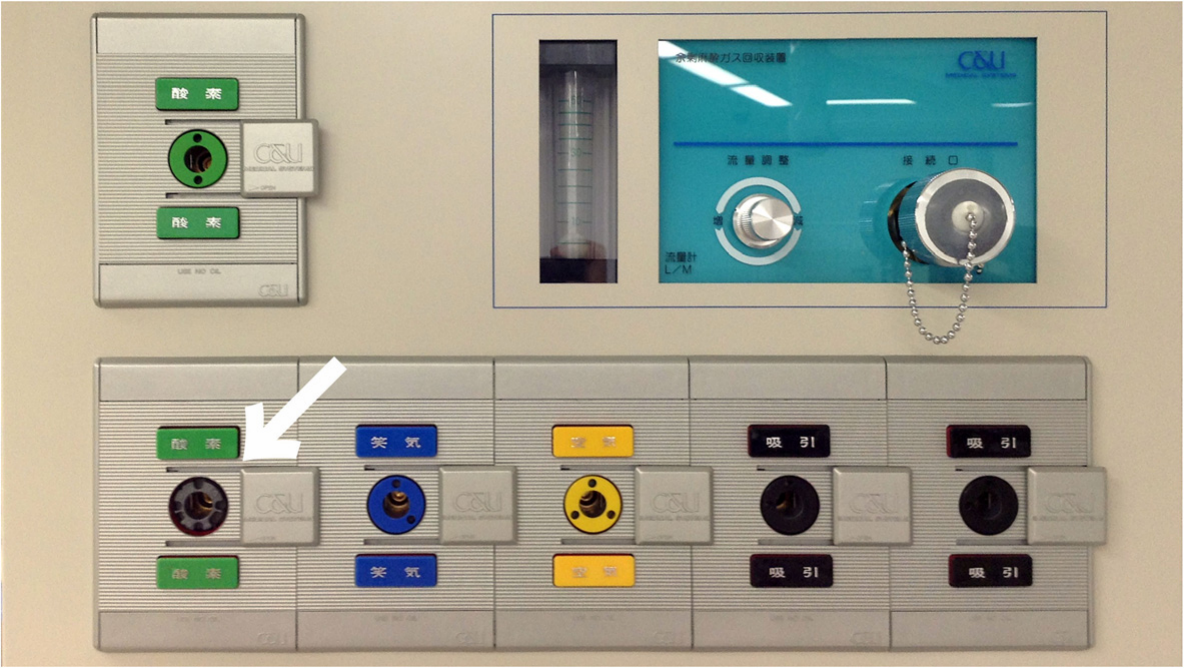
The ceiling-mounted pendant and the broken socket. The image shows the ceiling-mounted pendant installed in our operating room. The *arrow* indicates the pendant’s broken oxygen socket

During a morning check of an anesthesia machine in accordance with the guidelines published by the Japanese Society of Anesthesiologists, an anesthesiologist found that a gas-specific plastic pin guide that had been covering a universal gas outlet had disappeared (Fig. 2). Though procedures, such as a color-coding, a sequence of the socket, and a signage, would prevent an anesthesiologist from making an improper connection, a less-experienced medical worker could have connected the wrong pipeline to this outlet.

Despite the authors’ search efforts, the fallen cover could not be found. According to our interview to the anesthesiologists and the clinical engineers in our hospital at a later date, nobody noticed this situation. So, the proximate cause of the break is unclear. As only 8 months had passed since the operating room was built, the manufacturer had not made an annual periodic inspection. At a later date, engineers of the system’s manufacturer produced a specialized jig that was capable of grasping the outer periphery of the plastic pin guide and then measured the external vertical force required to dislodge it. This test revealed that the plastic pin guide could be dislodged by applying 29.4 N [18.6–54.175] (median [interquartile range]) of vertical force.

## Discussion

Wall-mounted pin-index medical gas pipeline systems have been sold in Japan since 1983. The design of such systems has barely changed since the start of their clinical use. According to the manufacturer, the incident described in this report was the first of its kind, and it was never suspected that such a situation could arise. In this system, there is no adhesive agent between the plastic (polyacetal) and metal components. Instead, three small ridges (height 0.3 mm, width 3.5 mm) inside the plastic component hook onto the metal base block. The required durability of such components is specified in JIS T 7101 (medical gas pipeline systems) based on International Standard Organization 9170-1 [2]. Although the manufacturer checked that the product met the JIS T 7101 regulations by repeatedly connecting and disconnecting gas pipes from the socket (10,000 times, using a machine), an integrated structure composed of a metal pin guide and socket is preferred for safe clinical use. The national standards provide regulations about the test of the gas-specificity in section 9.4.8 of JIS T 7101. They require that the manufacturer must try to connect each of the different gas pipelines sequentially. This test is inadequate for detecting to this type of malfunction. The national standards should be revised in this regard. Most of the products with a pin-index system have a metal socket with pin guides, unlike this product. Therefore, this error is the product-specific problem. It is our opinion that the design of the plastic component should be improved to prevent its unexpected detachment and that the design of the metal base block should be gas-specific. After discussion between authors and the manufacturer, they offered two solutions. First, they will make two small ridges around the outer periphery of the plastic pin guide, which is held by the front panel, so as not to dislodge. Second, they removed the versatility from the metal components. With these changes, the gas outlet remains foolproof if the plastic pin guide is lost.

## Conclusions

It is important to check that gas outlet systems are functioning in a gas-specific manner when they are used for clinical purposes. Every foolproof system does not always guarantee patient safety.

## Abbreviations

DISS, Diameter Index Safety system; JIS, Japan Industrial Standard
